# M153R Mutation in a pH-Sensitive Green Fluorescent Protein Stabilizes Its Fusion Proteins

**DOI:** 10.1371/journal.pone.0019598

**Published:** 2011-05-03

**Authors:** Yusuke V. Morimoto, Seiji Kojima, Keiichi Namba, Tohru Minamino

**Affiliations:** 1 Graduate School of Frontier Biosciences, Osaka University, Suita, Osaka, Japan; 2 Division of Biological Science, Graduate School of Science, Nagoya University, Chikusa-Ku, Nagoya, Japan; 3 Precursory Research for Embryonic Science and Technology, Japan Science and Technology Agency, Kawaguchi, Saitama, Japan; J. Craig Venter Institute, United States of America

## Abstract

**Background:**

Green fluorescent protein (GFP) and its fusion proteins have been used extensively to monitor and analyze a wide range of biological processes. However, proteolytic cleavage often removes GFP from its fusion proteins, not only causing a poor signal-to-noise ratio of the fluorescent images but also leading to wrong interpretations.

**Methodology/Principal Findings:**

Here, we report that the M153R mutation in a ratiometric pH-sensitive GFP, pHluorin, significantly stabilizes its fusion products while the mutant protein still retaining a marked pH dependence of 410/470 nm excitation ratio of fluorescence intensity. The M153R mutation increases the brightness *in vivo* but does not affect the 410/470-nm excitation ratios at various pH values.

**Conclusions/Significance:**

Since the pHluorin(M153R) probe can be directly fused to the target proteins, we suggest that it will be a potentially powerful tool for the measurement of local pH in living cells as well as for the analysis of subcellular localization of target proteins.

## Introduction

Green fluorescent protein (GFP) and related fluorescent proteins have been utilized to monitor and analyze a wide range of biological processes such as gene expression, protein localization and cell motility. These fluorescent proteins can also be used as the indicator of Ca^2+^ or ATP concentrations, or pH because they provide a high sensitivity in detection and are not toxic to living cells [1]–[3]. A GFP-derivative, pHluorin, is a ratiometric pH indicator with excitation wavelength at 410 and 470 nm and emission at 508 nm [3]. The relative emission intensity of pHluorin at the two excitation wavelengths show a remarkable pH dependence, thereby pH can be measured by the 410/470 nm excitation ratio, R_410/470_. The R_410/470_ ratio changes in less than 0.5 ms when pH is changed, indicating that the ratiometric measurement by pHluorin can detect a rapid pH change [4]. Since ratiometric methods, in which dual-wavelength measurements detect changes in the fluorescence absorption or emission spectra upon ion-binding, are independent of the concentration of the indicator, a precise and quantitative pH measurement of living cells can be easily carried out using pHluorin.

Since local pH is one of the most important parameters for probing the activities of live cells, pH imaging is becoming an efficient and useful method in various fields of biological sciences. Unlike GFP itself, however, GFP fusion proteins are fairly susceptible to proteolytic cleavage and so GFP is often released from target proteins, resulting in the poor signal-to-noise ratio of the fluorescent images. We therefore tried to improve the stability of fusion proteins in the experimental system that we study.

The flagellar motor of *Salmonella enterica* is powered by the electrochemical proton gradient across the cytoplasmic membrane. Two integral membrane proteins, MotA and MotB, form a proton channel to couple proton flow to torque generation. An interaction of MotA with a rotor protein FliG is required for torque generation [5]. The rotation-dependent proton influx has been estimated to be about 1,200 protons per revolution [6]. Since a decrease in intracellular pH significantly reduces flagellar motor rotation [7], [8], the proton release from the proton channel to the cytoplasm plays an important role in the torque generation process, and local pH near the motor must be tightly controlled. We therefore tried to measure local pH of the cytoplasmic side the motor by expressing pHluorin fusion proteins to flagellar motor proteins, such as pHluorin-FliG and pHluorin-MotB. However, the fusion proteins were susceptible to proteolytic digestion in the cell, making the high sensitivity pH imaging difficult.

In this study, we show that the M153R mutation in pHluorin markedly improves the stability of its fusion proteins while the mutant protein still retaining the pH dependence of 410/470 nm excitation ratio of fluorescence intensity to be useful as a pH sensor.

## Results

**Figure 1:**
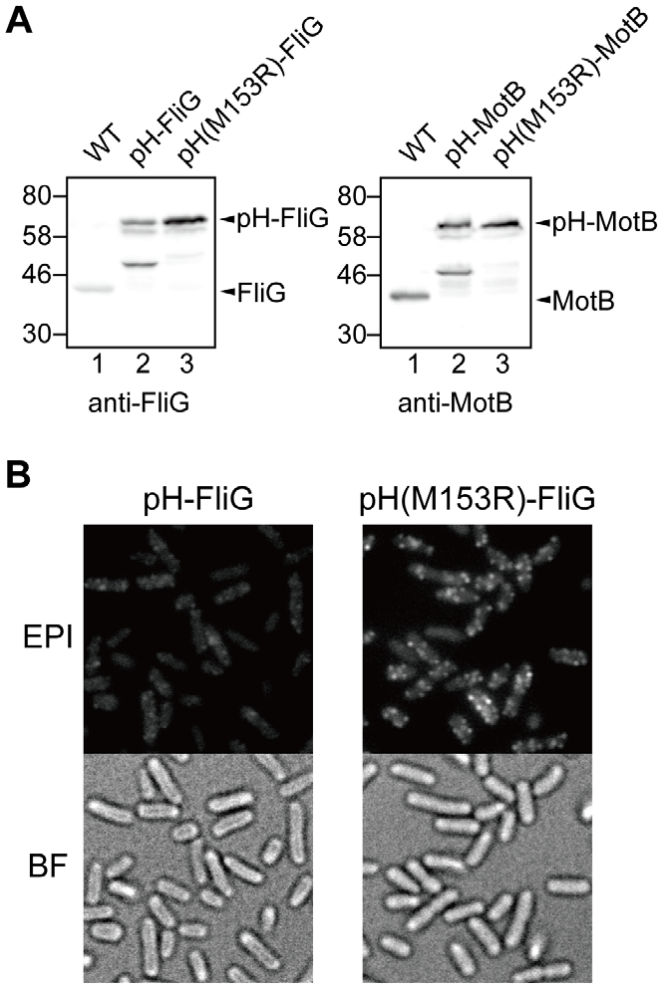
Effects of the M153R mutation in pHluorin on the protein stability of its fusion products. (**A**) Immunoblotting, using polyclonal anti-FliG (left panel) or anti-MotB antibody (right panel), of whole cell proteins prepared from SJW1103 (WT), YVM1002 (pHluorin-FliG, indicated as pH-FliG), YVM1004 (pHluorin(M153R)-FliG, indicated as pH(M153R)-FliG), YVM1001 (pHluorin-MotB, indicated as pH-MotB) and YVM1003 (pHluorin(M153R)-MotB, indicated as pH(M153R)-MotB). The positions of molecular mass markers (kDa) are shown on the left. (**B**) Fluorescence images (EPI) and bright field images (BF) of YVM1002 and YVM1004. The cells were grown overnight in LB at 30°C and observed by fluorescence microscopy.

To investigate local pH near the bacterial flagellar motor, the ratiometric pHluorin probe must be localized to the motor. Since it has been reported that the GFP-FliG and GFP-MotB fusion proteins are partially functional [9], [10], we fused pHluorin to the N-termini of FliG and MotB to produce *Salmonella pHluorin-fliG* and *pHluorin-motB* strains, respectively. However, these fusion proteins were unstable, and about a half of them were cleaved into a 47 kDa fragment as shown on the immunoblots (Figure 1A, lane 2 in both panels). Neither intact FliG nor MotB was observed, suggesting that the cleavage must occur within pHluorin. To identify the cleavage sites, a His_8_ tag was attached to pHluorin-MotB at its C terminus to facilitate protein purification. The cleavage product of pHluorin-MotB-His_8_ was purified by Ni-NTA affinity chromatography, and its molecular mass was measured by matrix-assisted laser desorption ionization time-of-flight mass spectrometry. The molecular mass was around 43.2–44.8 kDa. In search of possible proteolytic fragments having these masses, we identified possible cleavage sites to be at Met-153, Ala-154, Lys-162 or Ala-163 of pHluorin. Therefore, we carried out site-directed mutagenesis of these residues to see if any of such mutations have a stabilizing effect on the fusion proteins. The M153R mutation markedly stabilized both pHluorin-FliG and pHluorin-MotB (Figure 1A, lane 3 in both panels) while the other mutations showed no improvement (data not shown).

We next investigated whether the M153R mutation increases the signal to noise (S/N) ratio. Since the turnover of GFP-FliG between the cytoplasmic pool and functional motors does not occur [11], we analyzed the subcellular localization of pHluorin-FliG and pHluorin(M153R)-FliG by epi-illumination fluorescence microscopy. The M153R mutation substantially increased the number of fluorescent spots of pHluorin-FliG (Figure 1B). The fluorescence intensities of the pHluorin-FliG and pHluorin(M153R)-FliG spots were 1,065±278 A.U. (n = 109) and 3,560±1322 A.U. (n = 126), respectively, indicating that the M153R mutation resulted in a remarkable improvement in the S/N ratio of the fluorescent images.

**Figure 2:**
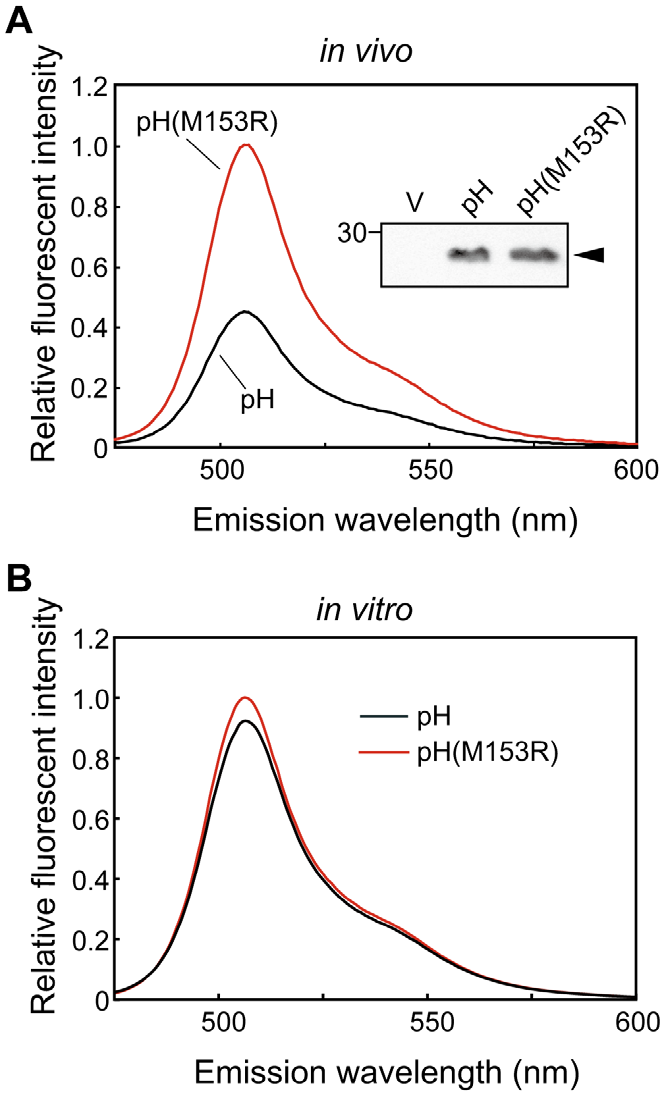
Effect of the M153R mutation on Ratio_410/470_. (**A**) Fluorescent intensities of SJW1103/pYC001 (pHluorin) and SJW1103/pYVM001 (pHluorin(M153R)) cells grown in T-broth at 30°C. Emission spectra with 395 nm excitation were measured by a fluorescence spectrophotometer. The measurements were done at 23°C. Inset: Immunoblotting, using polyclonal anti-GFP antibody, of whole cell proteins. (**B**) Fluorescent intensities of purified pHluorin and pHluorin(M153R).

To test whether the M153R mutation affects the brightness of pHluorin alone *in vivo*, we transformed a *Salmonella* wild-type strain, SJW1103, with a plasmid encoding pHluorin(M153R) on pKK223-3 and analyzed the fluorescent intensity with a spectrophotometer (Figure 2A). We used SJW1103 expressing pHluorin as a control. Immunoblotting with polyclonal GFP antibody revealed that the expression level of pHluorin(M153R) was the same as that of pHluorin (Figure 2A, inset) and wild-type GFP (data not shown), indicating that the M153R mutation does not alter the stability of pHluorin itself. Interestingly, the fluorescent intensity of pHluorin(M153R) was approximately 2.5-fold brighter than that of pHluorin. When the fluorescence intensities of purified pHluorin and pHluorin(M153R) were measured at the same protein concentration, there was no difference in the fluorescence intensity (Figure 2B). This result indicates that the M153R mutation does not increase the intrinsic brightness of properly matured pHluorin molecules. Therefore, we conclude that the M153R mutation improves the folding efficiency of pHluorin *in vivo*.

**Figure 3:**
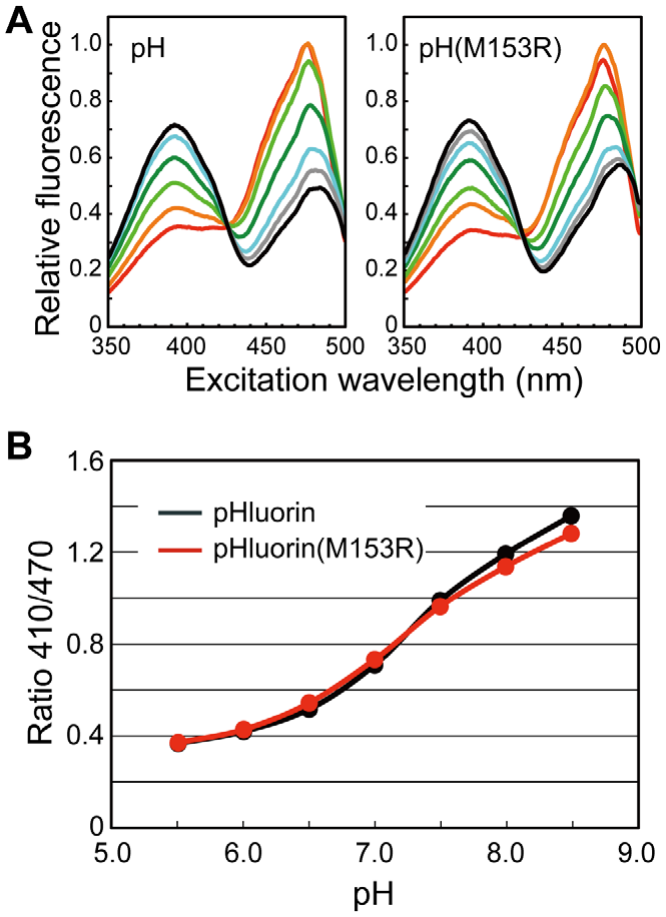
pH dependence of fluorescence excitation spectra and Ratio_410/470_ of pHIuorin and pHluorin(M153R). (**A**) Fluorescence excitation spectra. The different colored lines refer to different pH values. red, pH 5.5; orange, pH 6.0; light green, pH 6.5; green, pH 7.0; cyan, pH 7.5; grey, pH 8.0; black, pH 8.5. (**B**) Ratio_410/470_. The fluorescence excitation spectra of purified proteins were recorded on a fluorescence spectrophotometer. The measurements were done at 23°C.

It has been reported that the M153A mutation shifts the excitation wavelength of GFP(S65T) to a longer wavelength [12]. We therefore measured the excitation spectra of purified pHluorin and pHluorin(M153R) (Figure 3). The M153R mutation changed neither the excitation wavelengths nor the emission intensity ratios at the excitation wavelengths of 410 and 470 nm over a pH range from 5.5 to 8.5.

**Figure 4:**
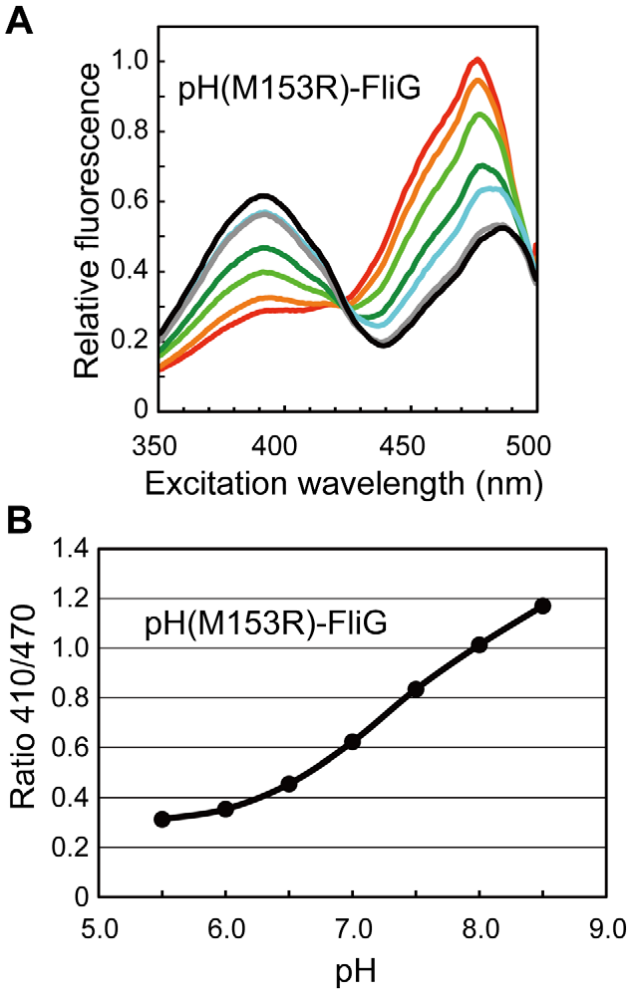
pH dependence of fluorescence excitation spectra and Ratio_410/470_ of pHluorin(M153R)-FliG-His_6_. (**A**) Fluorescence excitation spectra. The different colored lines refer to different pH values. red, pH 5.5; orange, pH 6.0; light green, pH 6.5; green, pH 7.0; cyan, pH 7.5; grey, pH 8.0; black, pH 8.5. (**B**) Ratio_410/470_. The fluorescence excitation spectra of the purified protein were recorded on a fluorescence spectrophotometer. The measurements were done at 23°C.

We next examined whether pHluorin(M153R)-FliG can be used as a ratiometric pH sensor. The R_410/470_ ratio of purified pHluorin(M153R)-FliG-His_6_ showed a pH-dependence from 0.3 at pH 5.5 to 1.2 at pH 8.5, which is as large as that of pHluorin(M153R) (Figure 4), indicating that pHluorin(M153R)-FliG can be a useful tool to measure local pH near the flagellar motor.

## Discussion

GFP has been used to determine subcellular protein localization. A peptide linker between GFP and the target protein is required for the stability and function of fusion proteins. However, GFP is often removed from fusion proteins by proteolytic cleavage. Here, we directly fused the ratiometric pHluorin probe to the N-termini of *Salmonella* FliG and MotB and found that these fusion proteins are also unstable *in vivo* (Figure 1A). As pHluorin itself is stable (Figure 2, inset), the fusion to target proteins presumably induces a conformational change in pHluorin, resulting in proteolytic cleavage of the fusion proteins. Site-directed mutagenesis revealed that the M153R mutation in pHluorin considerably improved the stability of its fusion proteins (Figure 1A). The M153R mutation also increased not only the number of fluorescent spots of pHluorin-FliG in *Salmonella* cells but also their fluorescence intensity, improving the S/N ratio of the images (Figure 1B). The M153R mutation did not change the 410/470-nm excitation ratios of pHluorin (Figure 3), indicating that pHluorin(M153R) can be used as a pH sensor. The 410/470-nm excitation ratios of pHluorin(M153R)-FliG-His_6_ also showed a similar pH-dependence (Figure 4). Since the pHluorin(M153R) probe can be directly fused to the target proteins, we believe that the pHluorin(M153R) probe can be a potentially powerful tool not only for the analysis of subcellular localization of target proteins but also for local pH measurement in living cells. We are currently developing a high-resolution pH imaging system using pHIuorin(M153R) as a probe.

## Materials and Methods

### Bacteria, plasmids, DNA manipulations and media

Bacterial strains and plasmids used in this study are listed in Table 1. Procedures for DNA manipulation were carried out as described previously [13]. L-broth, T-broth and motility medium were prepared as described [7]. Tc^S^ plates were prepared as described by Maloy and Nunn [14]. Ampicillin and tetracycline were added to LB at a final concentration of 100 µg/ml and 15 µg/ml, respectively.

**Table 1:** Strains and Plasmids used in this study.

Strains and Plasmids	Relevant characteristics	Source or reference
*E. coli*		
BL21(DE3) pLysS	T7 expression host	Novagen
*Salmonella*		
SJW1103	Wild type for motility and chemotaxis	[19]
YVM1001	*pHluorin-motB*	This study
YVM1002	*pHluorin-fliG*	This study
YVM1003	*pHluorin*(M153R)*-motB*	This study
YVM1004	*pHluorin*(M153R)*-fliG*	This study
YVMT001	*motB*::*tetRA*	This study
YVMT002	*fliG*::*tetRA*	This study
Plasmids		
pGST-pHluorin	pGEX2T/GST-pHluorin	[3]
pYC001	pKK223-3/pHluorin	[8]
pNSK22pH	pTrc99A/MotA+pHluorin-MotB-His_8_ *	This study
pNSK22pH(M153R)	pTrc99A/MotA+pHluorin(M153R)-MotB-His_8_ *	This study
pYVM001	pKK223-3/pHluorin(M153R)	This study
pYVM007	pGEX2T/GST-pHluorin(M153R)	This study
pYVM013	pTrc99A/pHluorin(M153R)-FliG-His_6_	This study

*In this pHluorin-MotB-His_8_ fusion construct, N-terminal 28 residues of MotB (Met1- Lys28) are attached to the N-terminus of pHluorin as described before [9].

### Construction of *Salmonella* strains expressing pHluorin-fusion proteins

To construct *Salmonella pHluorin-fliG*, and *pHluorin-motB strains*, *the fliG* or *motB* gene on the chromosome was replaced by the *gfp-fliG or gfp-motB* allele, respectively, by using the λRed homologous recombination system developed by Datsenko and Wanner [15]. First, the *tetRA* genes were inserted into the 5′-end of *fliG* or *motB* to create *fliG*::*tetRA* or *motB*::*tetRA*, respectively. Then, to replace the *tetRA* genes by pHluorin (GenBank accession No. AF058694), the pHluorin or pHluorin(M153R) gene was amplified by PCR using pYC001 or pYVM001 as a template and primers shown in Table 2. The PCR products were purified using a QIAquick PCR purification kit (QIAGEN). The *fliG*::*tetRA* or *motB*::*tetRA* strain transformed pKD46 [15], which has a temperature-sensitive replicon, was grown in 5-ml L-broth containing ampicillin and 0.2% L-arabinose at 30°C until OD_600_ had reached 0.6. The cells were washed three times with ice-cold H_2_O and suspended in 50 µl of ice-cold H_2_O. 50 µl of cells were electroporated with 100 to 200 ng of purified PCR products using 0.1-cm cuvettes at 1.8 kV. Shocked cells were incubated in 1 ml L-broth for 1 h at 37°C. Then one-half were spread onto Tc^S^ plates because tetracycline-resistant cells cannot grow in Tc^S^ plates [15] and incubated overnight at 42°C to remove pKD46. The constructs were confirmed by DNA sequencing. The *pHluorin-fliG, pHluorin(M153R)-fliG*, *pHluorin-motB*, and *pHluorin(M153R)-motB* alleles are placed under the control of their native promoters.

**Table 2:** Primers used for construction of *pHluorin-fliG* and *pHluorin-motB* strains.

Name	Sequence
pH-motB_Fw	5′-gcagtgagaaacccaaaccagcagcagacgactgaggaagcatgagtaaaggagaagaacttttc-3′
pH-motB_Rv	5′-ctgcggcgttttacgacgacaatgggatgagcctgattttttttgtatagttcatccatgccatg-3′
pH-fliG_Fw	5′-gcgcgtggtggcgctggtcattcgccagtggatgagtaacgatcatgagtaaaggagaagaacttttc-3′
pH-fliG_Rv	5′-ggtcatcaacaaaatgacgcttttatcggtaccgctaagattacttttgtatagttcatccatgccatg-3′

### Preparation of whole cell proteins and immunoblotting

*Salmonella* cells were grown overnight at 30°C in T-broth with shaking. Cell pellets were suspended in a SDS-loading buffer and normalized by cell density to give a constant amount of cells. After sodium dodecyl sulfate-polyacrylamide gel electrophoresis, immunoblotting with polyclonal anti-FliG, anti-MotB and anti-GFP antibodies was carried out as described previously [16].

### Purification of proteolytic products of pHluorin-MotB-His_8_

Membranes containing overproduced pHluorin-MotB-His_8_ fusion proteins were solubilized by 0.2% (w/v) of dodecylphosphocholine (Anatrace) and their proteolytic cleavage products were purified by Ni-NTA affinity chromatography as described [17]. Molecular mass of the cleavage products was analyzed by a mass spectrometer (Voyager DE/PRO, Applied Biosystems) as described [13].

### Site-directed mutagenesis

Site-directed mutagenesis was carried out using QuickChange site-directed mutagenesis method as described in the manufacturer's instructions (Stratagene). The mutations were confirmed by DNA sequencing.

### Fluorescence microscopy

Epi-fluorescence of pHluorin fusion proteins was observed by an inverted fluorescence microscope (IX-71, Olympus) with a 150× oil immersion objective lens (UApo150XOTIRFM, NA 1.45, Olympus) and an Electron-Multiplying Charge-Coupled Device (EMCCD) camera (C9100-02, Hamamatsu Photonics) as described before [10].

### Purification and Spectroscopy of pHluorin, pHluorin(M153R) and pHluorin(M153R)-FliG-His_6_

pHluorin and pHluorin(M153R) were purified as described before [8]. pHluorin-FliG-His_6_ was purified by Ni-NTA affinity chromatography as described [17]. Fluorescence excitation spectra of purified pHluorin, pHluorin(M153R), and pHluorin-FliG-His_6_ in buffers of defined pH were recorded on a fluorescence spectrophotometer (RF-5300PC, Shimadzu), and the 410/470-nm excitation ratios of pHluorin fluorescence intensities were determined at different pH values to generate a calibration curve as described previously [18].

For measurements of intracellular pH, wild-type cells carrying pYC001 or pYVM001 were grown with shaking in T-broth at 30°C until the cell density had reached an OD_600_ of 1.0. The cells were washed twice with motility buffer and resuspended in motility buffer. The cells were diluted 1∶100 into motility buffer, and the fluorescence excitation spectra of the cells were recorded on a fluorescence spectrophotometer. The 410/470-nm excitation ratios were calculated and converted to pH values based on the calibration curve previously generated.
